# Vascular inflammation in patients undergoing anti-CD19 chimeric antigen receptor-T cells for haematologic malignancies: a call for cardiovascular surveillance

**DOI:** 10.1093/eurheartj/ehaf513

**Published:** 2025-08-19

**Authors:** Massimiliano Camilli, Andrea Guarneri, Lucia Leccisotti, Federico Ballacci, Marcello Viscovo, Eugenio Galli, Luca Maggio, Lorenzo Tinti, Priscilla Lamendola, Stefan Hohaus, Simona Sica, Federica Sorà, Patrizia Chiusolo, Gaetano Antonio Lanza, Giovanna Liuzzo, Francesco Burzotta, Alessandro Giordano, Filippo Crea, Antonella Lombardo, Giorgio Minotti

**Affiliations:** Department of Cardiovascular and Pulmonary Sciences, Catholic University of the Sacred Heart, Rome, Italy; Department of Cardiovascular Medicine, Fondazione Policlinico Universitario A. Gemelli IRCCS, L.go A. Gemelli Rome 1-00168, Italy; Department of Radiological Sciences and Haematology, Section of Nuclear Medicine, Università Cattolica del Sacro Cuore, Rome, Italy; Unit of Nuclear Medicine, Fondazione Policlinico Universitario A. Gemelli IRCCS, Rome, Italy; Department of Radiological Sciences and Haematology, Section of Nuclear Medicine, Università Cattolica del Sacro Cuore, Rome, Italy; Unit of Nuclear Medicine, Fondazione Policlinico Universitario A. Gemelli IRCCS, Rome, Italy; Department of Cardiovascular and Pulmonary Sciences, Catholic University of the Sacred Heart, Rome, Italy; Dipartimento di Scienze di Laboratorio ed Ematologiche, Fondazione Policlinico Universitario A. Gemelli IRCCS, Rome, Italy; Dipartimento di Scienze Radiologiche ed Ematologiche, Università Cattolica del Sacro Cuore, Sezione di Ematologia, Rome, Italy; Dipartimento di Scienze di Laboratorio ed Ematologiche, Fondazione Policlinico Universitario A. Gemelli IRCCS, Rome, Italy; Department of Cardiovascular and Pulmonary Sciences, Catholic University of the Sacred Heart, Rome, Italy; Department of Cardiovascular Medicine, Fondazione Policlinico Universitario A. Gemelli IRCCS, L.go A. Gemelli Rome 1-00168, Italy; Department of Cardiovascular and Pulmonary Sciences, Catholic University of the Sacred Heart, Rome, Italy; Department of Cardiovascular Medicine, Fondazione Policlinico Universitario A. Gemelli IRCCS, L.go A. Gemelli Rome 1-00168, Italy; Dipartimento di Scienze di Laboratorio ed Ematologiche, Fondazione Policlinico Universitario A. Gemelli IRCCS, Rome, Italy; Dipartimento di Scienze Radiologiche ed Ematologiche, Università Cattolica del Sacro Cuore, Sezione di Ematologia, Rome, Italy; Dipartimento di Scienze di Laboratorio ed Ematologiche, Fondazione Policlinico Universitario A. Gemelli IRCCS, Rome, Italy; Dipartimento di Scienze Radiologiche ed Ematologiche, Università Cattolica del Sacro Cuore, Sezione di Ematologia, Rome, Italy; Dipartimento di Scienze di Laboratorio ed Ematologiche, Fondazione Policlinico Universitario A. Gemelli IRCCS, Rome, Italy; Dipartimento di Scienze Radiologiche ed Ematologiche, Università Cattolica del Sacro Cuore, Sezione di Ematologia, Rome, Italy; Dipartimento di Scienze di Laboratorio ed Ematologiche, Fondazione Policlinico Universitario A. Gemelli IRCCS, Rome, Italy; Dipartimento di Scienze Radiologiche ed Ematologiche, Università Cattolica del Sacro Cuore, Sezione di Ematologia, Rome, Italy; Department of Cardiovascular and Pulmonary Sciences, Catholic University of the Sacred Heart, Rome, Italy; Department of Cardiovascular Medicine, Fondazione Policlinico Universitario A. Gemelli IRCCS, L.go A. Gemelli Rome 1-00168, Italy; Department of Cardiovascular and Pulmonary Sciences, Catholic University of the Sacred Heart, Rome, Italy; Department of Cardiovascular Medicine, Fondazione Policlinico Universitario A. Gemelli IRCCS, L.go A. Gemelli Rome 1-00168, Italy; Department of Cardiovascular and Pulmonary Sciences, Catholic University of the Sacred Heart, Rome, Italy; Department of Cardiovascular Medicine, Fondazione Policlinico Universitario A. Gemelli IRCCS, L.go A. Gemelli Rome 1-00168, Italy; Department of Radiological Sciences and Haematology, Section of Nuclear Medicine, Università Cattolica del Sacro Cuore, Rome, Italy; Unit of Nuclear Medicine, Fondazione Policlinico Universitario A. Gemelli IRCCS, Rome, Italy; Department of Cardiovascular and Pulmonary Sciences, Catholic University of the Sacred Heart, Rome, Italy; Center of Excellence of Cardiovascular Sciences, Ospedale Isola Tiberina—Gemelli Isola, Rome, Italy; Department of Cardiovascular and Pulmonary Sciences, Catholic University of the Sacred Heart, Rome, Italy; Department of Cardiovascular Medicine, Fondazione Policlinico Universitario A. Gemelli IRCCS, L.go A. Gemelli Rome 1-00168, Italy; Unit of Drug Sciences, University Campus Bio-Medico, and Unit of Clinical Pharmacology, Fondazione Policlinico Universitario Campus Bio-Medico, Rome, Italy

**Keywords:** Cardio-Oncology, Chimeric Antigen Receptor T cell therapy, Cardiovascular Imaging, PET, Inflammation

**This paper was guest edited by Milton Packer**

## Introduction

Anti-CD19 chimeric antigen receptor (CAR)-T cell therapy has shown remarkable efficacy in CD19^+^ B-cell malignancies.^1,2^ While prospective data on the effects of CAR-T cells on myocardial function are accumulating,^3–6^ there is limited, uncertain information about possible vascular effects,^7^ which could well occur if one considered that CAR-T cell therapy is accompanied by cytokine release syndrome (CRS); accordingly, recent studies have shown that other immunologic therapies may aggravate systemic cardiovascular inflammation and the risk of cardiovascular events.^8^ This information clearly advocates for dedicated research in this field.

The present study sought to evaluate the usefulness of standard-of-care whole-body Positron emission tomography/computed tomography (PET/CT) with ^18^F-fluorodeoxyglucose (FDG) imaging for detecting changes of arterial and venous inflammation after anti-CD19 CAR-T cells infusion.

## Methods

Patients candidate for CAR-T cells therapy for relapsed or refractory (R/R) B-cell aggressive lymphomas were consecutively recruited from January 2022 to March 2024 in a single centre study at Fondazione Policlinico Universitario Agostino Gemelli IRCCS, Rome. All patients underwent ^18^F-FDG PET/CT before and 30 days after CAR-T cells infusion for oncologic purposes. Three anti-CD19 CAR-T cell formulations were used according to clinicians’ indications: Axicabtagene Ciloleucel, Tisagenlecleucel and Brexucabtagene Autoleucel. Routine blood analyses were performed during hospitalisation for CAR-T cells infusion and changes in inflammatory markers from baseline to 14 days after infusion were determined. The modified Endothelial Activation and Stress Index (mEASIX) score was calculated as follows: lactic dehydrogenase [LDH; U/L] × C-reactive protein [CRP; mg/dL]/platelets [PLTs; 10^9^ cells/L], as previously described.^9^

Maximum standardized uptake (SUVmax) of arterial and venous wall were calculated at baseline and 30 days after therapy. Target to background ratio (TBR) was obtained through the ratio between each SUVmax value and the SUVmean of blood pool in superior vena cava, as previously described.^9^ In order to incorporate both arterial and venous measurements, images were analysed by SUVmax rather than TBR. Furthermore, SUVmax was chosen since its high reproducibility, ease of calculation and because its measurement may not be affected by slight difference of ROI definition. Individual baseline predictors were evaluated by separate addition to the primary model.

To minimize biases, all images were obtained with the same scanner and according to a standard institutional acquisition protocol (see *Figure Legends* for details of image acquisition).

Response to CAR-T therapy was assessed using whole-body FDG PET/CT. Patients who achieved a complete response (CR) or partial response (PR) were classified as responders. CR and PR were defined as the complete disappearance or a reduction in the extent of all detectable signs of lymphoma, respectively. Patients with less than PR were classified as non-responders.

We further included a control group of 15 patients with diffuse large B-cell lymphoma (DLBCL) treated with autologous haematopoietic stem cell transplantation (HSCT) and matched with the CAR-T patients with respect to demographic and disease characteristics (median age 54 years old; 53% female; 33% non-responders). All patients received anthracycline-containing regimens prior to transplantation, the conditioning regimen was Fotemustine/Etoposide/Cytarabine/Melphalan, and PET-CT was done both before and after HSCT.

Continuous variables were reported as medians (IQR) and compared using Mann–Whitney *U* tests; categorical variables as counts (percentages) using Fisher's exact test. Inflammatory biomarker changes between responders and non-responders were assessed using Repeated-Measures ANOVA. Vascular SUVmax values were compared using paired *t*-tests and analysed longitudinally (before and after CAR-T therapy) with linear mixed-effects models including fixed effects for time, response type, and vessel subtype, using random effects to account for between-patient variability. For comparison with the HSCT cohort, a separate mixed-effects model incorporating fixed effects for time, response type, cohort (CAR-T vs. HSCT), and their interaction was used. Two-tailed *P*-values <.05 were considered statistically significant. Analyses were performed using R 4.4.2 software.

## Results

### Patients’ characteristics

**Figure 1:**
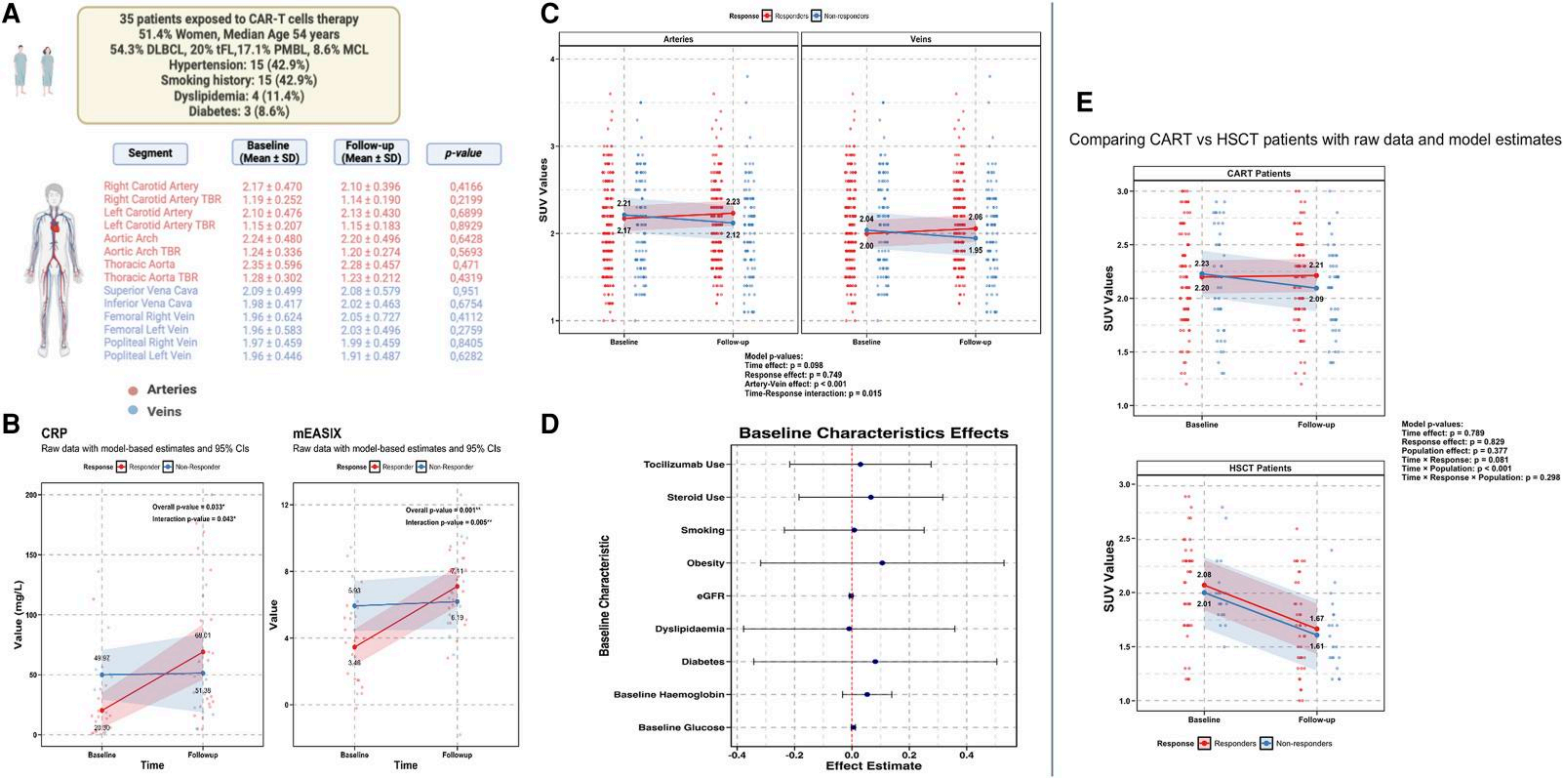
(*A*) ^18^F-FDG PET/CT imaging of arterial and venous vascular segments at baseline and 30 days after CAR-T cells infusion; (*B*) Inflammatory biomarkers (CRP and mEASIX) in responders (red) and non-responders (blue) at baseline and follow-up. Individual data points represent raw measurements, with model-predicted means (solid lines) and 95% confidence intervals (shaded areas). The repeated measures ANOVA revealed significant overall and time-response interaction effects for both CRP (*P* = .033, *P* = .043) and mEASIX (*P* = .001, *P* = .005). Responders showed lower baseline values but increased follow-up levels of inflammatory markers, while non-responders maintained relatively stable values. (*C*) Vascular inflammation (SUVmax values) across arteries and veins in responders (red) and non-responders (blue) at baseline and follow-up. Individual data points represent raw data points, with model-predicted means (solid lines) and 95% confidence intervals (shaded areas). The mixed-effects model revealed a significant artery-vein effect (*P* < .001) and time-response interaction (*P* = .015). Responders showed increased SUVmax values at follow-up. (*D*) Correlation between baseline patients’ characteristics, pharmacological therapies for Cytokine Release Syndrome, and SUVmax values. Plot displays the beta coefficients (effect estimates) for the baseline characteristics as dots with horizontal lines representing their 95% confidence intervals. The vertical red dashed line at zero represents the null effect. (*E*) Comparison of changes in vascular SUVmax values between CAR-T and HSCT patients, using a mixed effects model. Distinct patterns of vascular inflammation are shown. HSCT patients exhibited a significant decrease in SUVmax values at follow-up regardless of response type (*P* < .001), while the CAR-T patients maintained overall stable values with persistent significant differences between responders (red) and non-responders (blue). *Acquisition protocol:* Patients were instructed to fast for at least 6 h prior to FDG administration, which resulted in pre-FDG glucose serum levels of <120 mg/dL. All patients were given an FDG activity of 3–4 MBq/Kg body weight; PET/CT acquisition was performed 60 ± 5 min after FDG injection, from the skull base to the knees and with the arms supported above the head. After normalisation and correction for photon attenuation, dead time, randoms and scatters, PET data were reconstructed using an iterative algorithm (ordered-subsets expectation maximisation, 2 iterations and 21 subsets), with the combined effect of point spread function (PSF) modelling and time of flight (TOF). Slide thickness was 3 mm. Both baseline and post-CAR-T PET scans were performed with the same 3D PET/CT scanner. CAR-T, chimeric antigen receptor-T; CRP, C-reactive protein; DLBCL, Diffuse large B-cell Lymphoma; mEASIX, modified Endothelial Activation and Stress Index; MCL, Mantle Cell Lymphoma; SUVmax, maximum standardized uptake values; tFL, transformed Follicular Lymphoma; ^18^F-FDG PET/CT, Positron emission tomography/computed tomography (PET/CT) with 18F-fluorodeoxyglucose (FDG); PMBL, Primary Mediastinal B Lymphoma

Thirty-five patients with R/R CD19^+^ B-cell lymphoma were included in this study (*Figure 1*), of whom 51% were females. Median age was 54 years (IQR: 42.5–62.0). The most used CAR-T cell formulation was Axicabtagene ciloleucel (57.1% of patients). At baseline, creatinine was 0.7 mg/dL (IQR: 0.6–0.9), glycaemia was 93 mg/dL (IQR: 86.7–100.5) and haemoglobin was 9.9 g/dL (IQR: 9.0–11.1). All patients were previously exposed to anthracycline-based regimens [doxorubicin median cumulative dose: 430 mg/m^2^ (IQR: 362–480)]; no patient had history of myocardial infarction or other atherothrombotic events. Twenty-five subjects (71.4%) experienced grade ≥2 CRS, requiring Tocilizumab in 65.7% of cases; corticosteroids were used in 31.4% of subjects. At baseline, CRP and mEASIX score were higher in non-responders compared with responders [49.4 mg/dL (23.3–55.9) vs. 12.3 mg/dL (3.5–22.4), *P* = .02, and 6.5 (4.7–7.5) vs. 3.82 (1.5–5.2), *P* = .017, respectively]. No other significant differences were observed between groups in terms of baseline clinical or laboratory characteristics.

### Vascular wall SUVmax before and after CAR-T therapy

Segment-by-segment comparisons did not show significant differences in arterial or venous wall SUVmax between baseline and 30-days follow-up, when data from the whole population were examined (*Figure 1A*).

### Changes in inflammatory markers before and after CAR-T therapy

Baseline CRP levels were higher in non-responders than in responders [50.0 mg/L (SE: 11.5) vs. 20.3 mg/L (SE: 5.7)]. Following CAR-T infusion, CRP increased substantially in responders to 72.9 mg/L (SE: 13.4), but only marginally in non-responders to 61.4 mg/L (SE: 17.4). Repeated-measures ANOVA revealed a significant time-to-response interaction (*F* = 4.47, *P* = .044). Similarly, mEASIX score increased from baseline to follow-up in responders, from 3.46 (SE: 0.44) to 6.96 (SE: 0.39), while it showed minimal changes in non-responders, from 5.93 (SE: 0.78) to 6.46 (SE: 1.01). A significant time-to-response interaction was found (*F* = 9.55, *P* = .005). *Figure 1B* illustrates these differential patterns using model-based estimates with 95% confidence intervals.

Of note, there were no statistically significant changes in interleukin (IL)-6 levels, IL-2 receptor levels and peripheral blood expansion of CAR-T cells (not shown).

### Influence of patients’ characteristics, vessel type, and treatment response on vascular wall SUVmax

A linear mixed-effects regression model was fitted to predict segment-specific SUVmax value changes from baseline to follow-up, accounting for vessel type (artery vs. vein) and response to CAR-T cells therapy. Vessel type showed a statistically significant main effect, with veins exhibiting lower mean SUVmax values compared with arteries [*β* = −0.18, 95% CI: (−0.27, −0.08), *P* < .001].

Interestingly, the interaction effect between time and response was statistically significant [*β* = −0.15, 95% CI: (−0.27, −0.03), *P* = .015] (*Figure 1C*), indicating that the change in vascular SUVmax from baseline to follow-up significantly differed between responders and non-responders. Responders showed an increase in SUVmax values over time, while non-responders exhibited a decrease. These findings were not influenced by patients’ baseline characteristics and therapy used (*Figure 1D*).

### Comparison between CAR-T and HSCT patients

Lymphoma patients undergoing HSCT showed distinct patterns of vascular inflammation when compared with CAR-T patients. In contrast to the response-dependent pattern observed in CAR-T patients, those undergoing HSCT exhibited a significant decrease in SUVmax at follow-up, regardless of the response type (interaction between time and population: [*β* = −0.42, 95% CI: (−0.59, −0.25), *P* < .001], as shown in *Figure 1E*.

## Discussion

Our study shows limitations including small sample size, retrospective design, and short follow-up. The pathophysiologic significance of baseline vascular inflammation and its possible connections with tumour burden and patient’s immunity merit explorations. Having recognized these limitations, our report provides novel evidence for early effects of CAR-T cells infusion on vascular inflammation as detected by ^18^F-FDG PET/CT imaging in patients with R/R B-cell lymphomas. In particular, whereas no changes in baseline and post-CAR-T cells SUVmax values could be detected in the overall population, patients responding to CAR-T cells showed higher vascular inflammation at 30 days compared with non-responders. Compared with the CAR-T group, patients undergoing HSCT showed a significant SUVmax decrease over time in both responders and non-responders. These results may suggest a unique CAR-T cells-induced pattern of vascular inflammation compared with other cell-based therapies.

The acute hyperinflammatory response associated with CAR-T cells infusion may well support our interpretation of a persistent/residual vascular inflammation process. Other explanations, possibly involving non-neoplastic processes of hypercaptation, such as immune reconstitution, may appear less convincing, as they would be expected to localize more in primary or secondary lymphoid organs than in vascular walls.

Responders also presented a substantial increase of both CRP and mEASIX score, markers of inflammation and endothelial dysfunction,^9^ which corroborates a pivotal role of cytokine release and systemic inflammation in the pathogenesis of cardiovascular toxicity induced by CAR-T cells.^4,6,7^

The pathophysiology of thrombosis and atherosclerosis is closely linked with inflammation, through several mechanisms like vascular injury, endothelial dysfunction and lipid peroxidation.^10^ Inflammation introduces a residual, non-modifiable risk of long-term cardiovascular events, recently addressed in clinical trials.^10^ Our results, if confirmed in larger prospective studies and other tumour types, may help refine our appraisal of cardiovascular risk associated with new immunotherapies, in particular CAR-T cells therapy, broadening our focus from left ventricular function to the cardiovascular system as a whole. As well, adding vascular inflammation in the clinical evaluation of patients may help identify subjects at higher risk of long-term events and tailor surveillance/preventive strategies.

## Declarations

### Data Availability

The data underlying this article will be shared on reasonable request to the corresponding author.

### Pre-registered Clinical Trial Number

The pre-registered clinical trial number is: ID 4879 Prot 0020777/22 Amendment 02/2024.
